# A Case Report of Iliopsoas Abscess Secondary to Small Bowel Fistula

**DOI:** 10.7759/cureus.34749

**Published:** 2023-02-07

**Authors:** Veronica Guerrero, Agnes Park, Steven Y Zhou

**Affiliations:** 1 General Surgery, Northwestern Medicine McHenry Hospital, McHenry, USA; 2 General Surgery, Chicago Medical School, North Chicago, USA

**Keywords:** small bowel obstruction, atypical back pain, incision and drainage of abscess, fistulization, iliopsoas abscess

## Abstract

Iliopsoas abscesses (IPAs) are rare infections in the musculature that can be difficult to diagnose due to nonspecific presentations. These abscesses are most commonly caused by either the hematogenous spread of a separate infectious source in the body or secondary to Crohn’s disease and are typically treated with antibiotic therapy and percutaneous drainage. For cases complicated by bowel disease, multiloculated psoas abscess, or gas-forming organisms, surgical drainage may be indicated. We present the case of an 81-year-old female with a history of colon cancer status post-cecum resection who presented with back pain, thigh pain, and constipation. Computerized tomography imaging showed concurrent small bowel obstruction and a right IPA extending down to the right thigh. Laparoscopic exploration revealed a small bowel fistulization to the right iliopsoas as the source of infection. Resection of the small bowel and surgical incision and drainage of the abscess were necessary for her treatment. The patient was discharged with vacuum-assisted closure of her wound after a hospital course complicated with chronic diarrhea. Bowel fistulization should be considered a potential cause of IPAs in patients with a complicated gastrointestinal history.

## Introduction

Iliopsoas abscesses (IPAs) are a rare condition of a suppurative collection forming within the iliacus and psoas muscles due to bacterial infection. IPA can be divided into primary and secondary. Primary IPA is caused by hematogenous spread from an infectious source in the body, and secondary IPA is caused by an underlying disease, most commonly Crohn’s disease, with increased risk in those who undergo procedures in the groin, lumbar, or hip area [1]. They occur more commonly in men than women and younger than older individuals [2,3].

IPA presents with vague clinical features including back or flank pain, fever, inguinal mass, limp anorexia, and weight loss [1]. Only 30% of patients with IPA present with the psoas-muscle signs of fever, flank pain, and limitation of hip movement [4]. Definitive diagnosis is obtained with computerized tomography (CT) but magnetic resonance imaging (MRI) may be superior for the visualization of soft tissues and abscess walls [5]. Treatment involves antibiotics and drainage of the abscess. Drainage should be done percutaneously unless caused by bowel disease requiring resection, the presence of multiloculated psoas abscess, or the presence of gas-forming organisms [6]. A culture of the abscess is required to determine the appropriate antibiotics, but empiric antibiotics should be initiated prior to culture results. Mortality is reported to be 2.4% in primary IPA and 19% in secondary IPA with proper treatment [5]. Here, we describe a patient with a right IPA secondary to a small bowel fistulization to the right iliopsoas muscle, who had rare complications of small bowel obstruction (SBO) and abscess extending to the inner upper thigh requiring surgical incision and drainage (I&D) and antibiotic therapy.

## Case presentation

An 81-year-old female with a past medical history of colon cancer in remission, osteopenia, iron deficiency anemia, chronic kidney disease stage 3A, hypertension, hyperlipidemia, and obesity presented to the emergency department due to five days of worsening back and leg pain. Her status was post-cecum resection and radiation since 2017 for her colon cancer. Her pain was described as “sciatic,” radiating from her right lower sacrum to her right knee, and was associated with occasional tingling. She reported anorexia and difficulty ambulating due to her pain. She denied urinary or fecal incontinence, recent fevers, chills, or nausea.

On examination, she was afebrile with a blood pressure of 95/65 mmHg consistent with her historic measurements, respiratory rate of 16/minute, and pulse of 86/minute. Her physical examination was unremarkable, with a normal musculoskeletal (MSK) examination, no neurologic deficits, normal bowel sounds, and no gross edema.

Laboratory studies showed leukocytosis (leukocytes 32 × 10^3^/µL, neutrophils 85%, lymphocytes 1%, monocytes 2%, bands 12%). MRI of the lumbar spine with and without contrast and X-ray of the lumbar spine both confirmed anterolisthesis of L5 on S1. Chest X-ray was unremarkable. Blood cultures were negative.

**Figure 1:**
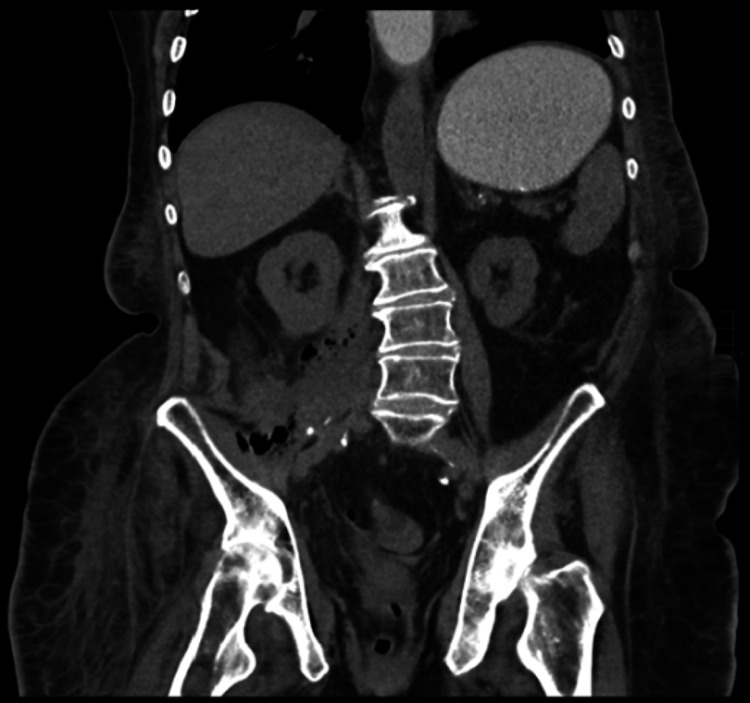
CT of the abdomen without contrast showing air, fluid, and abnormal thickening of the right iliopsoas musculature extending down to the right thigh, concerning for abscess.

**Figure 2:**
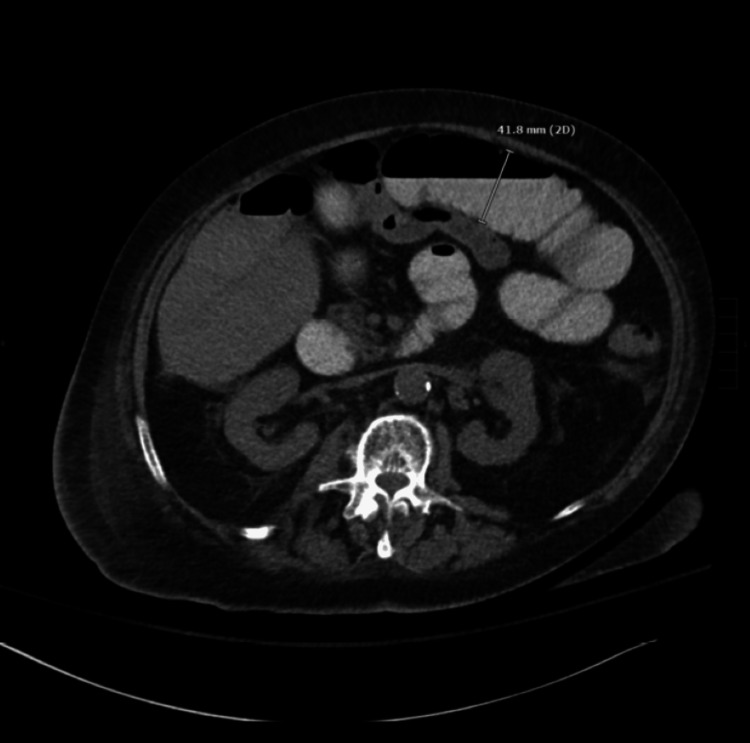
CT of the abdomen without contrast showing dilatation of the small bowel loops due to small bowel obstruction.

**Figure 3:**
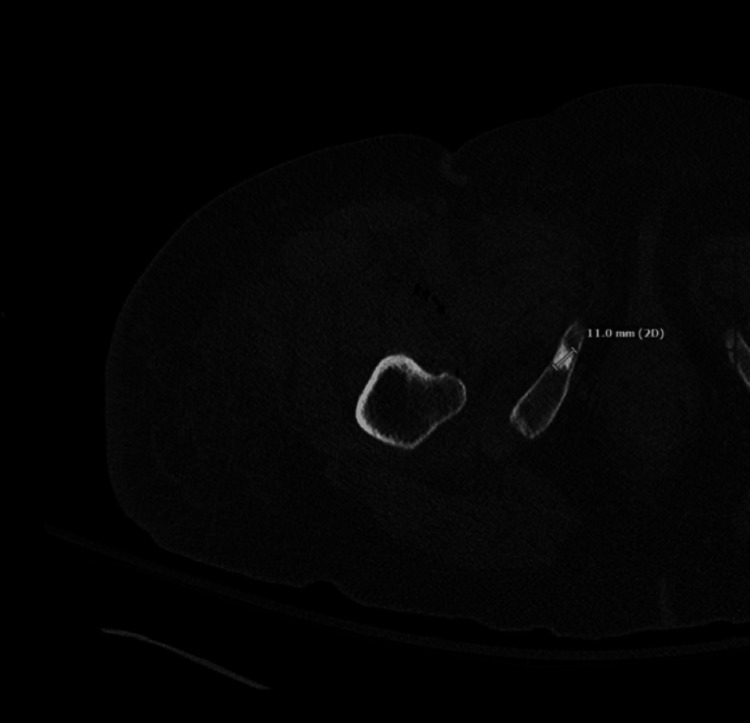
CT of the right lower extremity without contrast showing inflammatory changes with soft-tissue air tracking from the right iliopsoas musculature to the right mid-quadriceps musculature. No drainable, well-defined fluid collection was identified.

The patient’s unexplained leukocytosis in conjunction with her pain prompted further imaging. CT scan of her abdomen and pelvis without contrast revealed air and fluid tracking along the right iliopsoas muscle with possible fasciitis of the right upper thigh (Figure 1). Despite the patient exhibiting no obvious symptoms, the same imaging also confirmed SBO along the right iliopsoas muscle and at the bowel containing a ventral hernia (Figure 2). Extension to the right upper thigh was confirmed with a CT scan of the right lower extremity without contrast (Figure 3). She was started on empiric intravenous (IV) piperacillin-tazobactam 3.375 g q8 hours, and a nasogastric tube (NGT) was placed on low intermittent suction.

The patient’s condition did not show signs of improvement despite several days of antibiotic and NGT treatment. Repeat laboratory studies were performed on day six of admission to assess if further intervention was necessary. Worsening leukocytosis was noted, and on day eight, a CT-guided right psoas abscess drainage was successfully performed.

On day 13, an exploratory laparotomy was performed for SBO and to identify the source of the abscess. Exploration showed a small bowel fistula near the anastomosis to the posterior abdominal wall deep in the right iliopsoas muscle. Enteric-like drainage was found at this fistula leading to the anterior superior iliac spine, which was irrigated and suctioned. The friable and dilated open loops of the small bowel and a portion of the colon were divided and resected using a linear stapler and LigaSure for the mesentery. A side-to-side anastomosis was then performed between the small bowel and colon via an Endo GIA 60 mm, and the common channel was closed with a 60 mm stapler. The defect was brought together with a 3-0 silk running suture. A long Jackson-Pratt (JP) drain was placed in the right psoas abscess cavity. An additional JP drain was placed in the right upper quadrant of the abdomen after irrigation with copious amounts of saline.

Postoperatively, the patient developed worsening 10/10 right thigh pain associated with a patch of erythema on the anterior thigh, numbness and tingling, and difficulty ambulating. On postoperative day seven, bedside I&D was performed on the right thigh with 600 cc of feculent fluid suctioned. The thigh cavity tracked cephalad and was probed to break all loculations. Cultures were obtained which revealed 4+ white blood cells, 1+ yeast, 1+ gram-negative rods, and 1+ gram-positive cocci. During the following six days, over 600 cc of feculent fluid was suctioned from the right thigh. Continuous production of the feculent fluid prompted surgical I&D of the right thigh abscess.

The patient was prepped for I&D 16 days post-laparotomy. An elliptical incision measuring 4 × 5 cm was made to widen the existing bedside incision. Suction brought out copious amounts of purulence, and the right thigh abscess cavity was revealed to be over 15 cm deep toward the abdomen. Suction Yankauer was used to break up loculations. Significant slough fibrinous exudate throughout the base of the wound was debrided using a retractor and pickups, but the proximal side of the wound toward the abdomen and the iliopsoas was unable to be debrided due to the depth of the wound. The wound was irrigated and suctioned, and a vacuum-assisted closure of her wound (wound VAC) was placed.

Post-I&D recovery was complicated by abdominal pain, murky wound VAC drainage, and intermittent severe diarrhea. Abdominal pain resolved over the following weeks. Wound VAC drainage gradually improved, turning from murky to serosanguinous post-I&D day seven. Severe diarrhea was treated with various combinations of stool hardeners such as Imodium, cholestyramine, liquid codeine, and fiber. The patient was ultimately discharged with her wound VAC post-I&D day 16 after CT showed no signs of enteric fistula with stable findings in the pelvis and right thigh.

## Discussion

Small bowel fistula is a rare postoperative complication after colon surgery, causing pain and suffering to patients [7]. In addition, this complication has been associated with negative economic impact, increased morbidity, extended postoperative hospital stay, readmission, sepsis, and death [8].

A small bowel fistulization to the iliopsoas muscle in conjunction with SBO appears to be a rare pathology that is difficult to diagnose. IPA presents with vague clinical findings such as back or flank pain, fever, and weight loss, with only 30% of patients presenting with psoas-muscle signs [1,4]. IPA risk factors include having an infectious source in the body, underlying disease (Crohn’s disease), or recent procedures in the groin, lumbar, or hip area [1]. Our patient’s disease presentation deviated significantly from a typical presentation of psoas abscess, as the patient did not present with classic MSK signs (psoas sign) or constitutional symptoms such as fever, chills, and nausea [9]. The leukocytosis on laboratory work for this patient proved most valuable in identifying an infectious cause for her back and thigh pain.

CT imaging without contrast of the abdomen, pelvis, and lower extremities confirmed her diagnosis. In an emergent case, either CT imaging or ultrasound may be used to confirm the diagnosis [10]. However, in addition to being more precise than ultrasound imaging in detecting abscesses, CT may also provide a clearer view of abscess size, shape, and location for operative treatment [11]. MRI is superior to CT imaging for intraperitoneal abscess detection, but given its limited availability and complexity, MRI may be less suitable for unstable patients [9].

For patients with signs of IPA, a thorough gastrointestinal (GI) history may elucidate source identification. For patients with colorectal cancer, previous history of obstruction, Crohn’s disease, and previous GI surgery, fistulization of the bowel should be considered a potential source of infection, and surgical exploration may be warranted in patients without other obvious sources of infection [12].

A JP drain in the abscess cavity did not prove sufficient for the overall management of this patient’s abscess. Additionally, the patient complained of continued thigh pain and erythema. Upon I&D in the operating room, the abscess was revealed to be significantly larger than expected, likely causing her continuous feculent drainage at the bedside and her other constellation of symptoms. While traditional therapy for iliopsoas abscess is percutaneous drainage, this complex case necessitated surgical drainage [9]. The risk-benefit ratio of surgical intervention for the patient must be carefully considered, as surgical drainage of abscesses has a higher risk of complications, including a higher mortality rate [13].

## Conclusions

For patients with IPAs, initial symptoms can be vague, and patients may not present with obvious constitutional symptoms associated with infection. In patients with back pain, IPAs, while rare, should be considered in differential diagnoses. Initial labs showing significant leukocytosis may help identify these abscesses early. If no other source of infection can be identified clinically, CT imaging can be useful in locating these abscesses. For large muscle abscesses resistant to medical management, surgical I&D should be considered. Patients with a complicated GI history and confirmatory imaging for IPAs should have bowel fistulization as a potential source for their infection.
